# Diagnostic value of D2-40 immunostaining for malignant mesothelioma: a meta-analysis

**DOI:** 10.18632/oncotarget.19041

**Published:** 2017-07-06

**Authors:** Chao He, Bo Wang, Chun Wan, Ting Yang, Yongchun Shen

**Affiliations:** ^1^ Department of Laboratory Medicine, West China Hospital of Sichuan University, Chengdu 610041, China; ^2^ Intensive Care Unit, West China Hospital of Sichuan University, Chengdu 610041, China; ^3^ Department of Respiratory and Critical Care Medicine, West China Hospital of Sichuan University and Division of Pulmonary Diseases, State Key Laboratory of Biotherapy of China, Chengdu 610041, China

**Keywords:** malignant mesothelioma, D2-40, diagnosis, meta-analysis

## Abstract

Malignant mesothelioma (MM) has become a global disease burden for its rising incidence and invariable fatality. D2-40 has been widely used as an immunostaining marker of diagnosing MM, while its diagnostic value has not yet been evaluated. Our study aimed to assess the overall accuracy of D2-40 immunostaining for diagnosing MM through a meta-analysis. A total of 22 studies with 2,264 participants were identified from PubMed, EMBASE, Web of Science, Scopus and the Cochrane database. The pooled sensitivity and specificity of D2-40 for MM was 0.86 (95% CI: 0.84–0.89) and 0.77 (95% CI: 0.74–0.79), respectively. The area under the summary receiver operating characteristic curve is 0.93, with a diagnostic odds ratio 40.37 (95% CI: 19.97–81.61). None of the study variates was found to be a source of heterogeneity after meta-regression analysis. In conclusion, D2-40 immunostaining may not give sufficient evidence by itself to diagnose MM and should be in combination with other markers to improve the accuracy of diagnosis.

## INTRODUCTION

Malignant mesothelioma (MM) is a highly aggressive carcinoma usually involving the pleura or peritoneum. Between 1994 and 2008, a total of 92,253 mesothelioma deaths has been reported to the World Health Organization from 83 countries, and the crude and age-adjusted mortality rates of mesothelioma were estimated as 6.2 and 4.9 per million population, respectively [1]. Moreover, an incidence peak was expected around 2020 according to the widespread exposure to asbestos [2]. Unfortunately, the patients suffering from MM showed poor response to the drug, and their median survival period was only 8 months (range 1∼69 months) [3].

The diagnosis of carcinoma is usually based on histopathological examination on tissue or cytological specimens. Because morphological features of MM mimic those of a variety of other carcinomas, immunohistochemical markers have been used to improve the diagnostic performance, as recommended by International Mesothelioma Interest Group [4]. Whereas, several follow-up studies reported that the misdiagnosis rate of MM was high (10% ∼44%) [5–7], even much higher (65%) in its pleural localization [8]. So, it is necessary to evaluate the diagnostic accuracy of the markers.

D2-40 is a commercial monoclonal antibody directed against the M2a antigen, which is associated with testicular and extratesticular seminomas and intratubular germ cell neoplasms [9]. Also, D2-40 is a marker for lymphatic endothelium and derived carcinomas [10]. In the past decade, D2-40 has been widely used as an immunostaining marker of diagnosing MM [4, 11–14]. However, the studies about its diagnostic accuracy obtained inconsistent results. Therefore, our study aimed to assess the overall accuracy of D2-40 immunostaining for diagnosing MM through a meta-analysis.

## MATERIALS AND METHODS

### Literature search and study selection

We conducted an independent literature search to identify the relevant studies in PubMed, EMBASE, Web of Science, Scopus and the Cochrane database up to December 31, 2016. The search terms were “Mesothelioma”, and “D2-40”, and “sensitivity or specificity or accuracy”. The reference lists of eligible studies were also manually searched to find the potential studies.

The included studies should meet all the following criteria: (1) it was an original study using D2-40 immunostaining to diagnose MM; (2) there was a diagnostic standard for MM; (3) there were sufficient data to generate a 2 × 2 table for calculating sensitivity and specificity; (4) it was published in English; (5) it involved at least 10 participants for case or control group.

Two independent researchers (CH and BW) screened and selected the eligible articles. Any disagreement between the two researchers was resolved by consultation with a third researcher (YCS).

### Data extraction and quality assessment

Two researchers (CH and BW) manually extracted the following data from each study: author; publication year; research country; participant; specimen; specimen preparation method; D2-40 antibody (clone, dilution, source); cutoff of positive immunostaining; the number of true-positive, false-positive, true-negative, and false-negative results; study design; and blinding. At the same time, the methodological quality of these studies was evaluated using the Quality Assessment for Diagnostic Accuracy Studies (QUADAS) score [15]. The discrepancies were resolved through consultation with a third researcher (YCS).

### Statistical analyses

The following parameters of diagnostic accuracy, together with their 95% confidence interval (95% CI), were calculated: sensitivity, specificity, positive/negative likelihood ratio (PLR/NLR), and diagnostic odds ratio (DOR). Forest plots for sensitivity and specificity were constructed. Summary receiver operating characteristic (SROC) curve was generated, and area under the cure (AUC) was calculated to assess the overall diagnostic performance. Publication bias was tested using Deeks’ funnel plots [16].

Potential heterogeneity among included studies was evaluated using the I^2^ inconsistency test. I^2^ > 50% suggested substantial heterogeneity, which was subsequently analyzed through a meta-regression analysis to determine possible sources of heterogeneity among the studies.

All the statistical analysis was completed by Meta-DiSc XI (Cochrane Colloquium, Barcelona, Spain) and STATA 12.0 (Stata Corporation, TX, USA) software. All statistical tests were two-sided, and statistical significance was set at *P* < 0.05.

## RESULTS

### Characteristics of included studies

**Figure 1:**
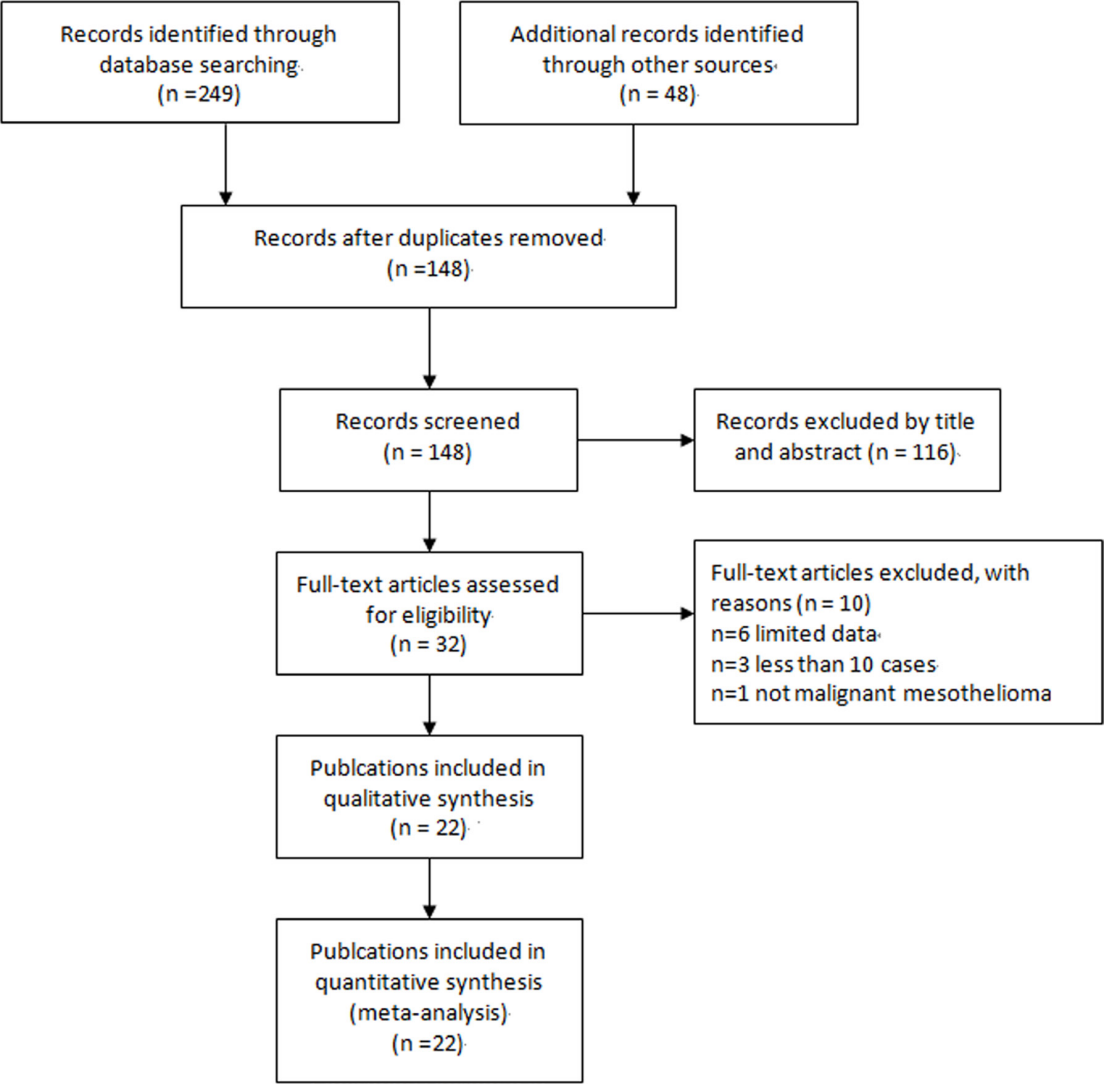
Flow diagram of literature search

A total of 22 studies were identified according to the inclusion criteria in our meta-analysis [5, 8, 17–36]. The process of selecting studies was shown in Figure 1. The characteristics of these studies were listed in Table 1. Overall, these studies originated from 8 countries, and involved 2,264 participants comprising 862 MM cases and 1,402 controls. The sample size of these studies varied from 20 to 282, with an average size of 103 participants. Lung carcinoma (778/1402, 55.5%) was the predominating control group (Supplementary Table 1). Four studies performed D2-40 immunostaining of cell blocks from the effusions [17–20] and the remaining 18 studies assayed the tissues from surgical section, biopsy and/or autopsy [5, 8, 21–36]. As shown in Table 2, 20 studies had QUADAS scores ≥ 9, suggesting the reliability of our meta-analysis results.

**Table 1:** Characteristics of included studies

Author (Ref)	Year	Country	Participants	Specimen	Specimen preparation	Clone	Dilution	Source	Cut-off
Bassarova AV [17]	2006	Norway	282	PE,PTE	Cell block	D2-40	1:200	Dako	Membranous staining
Saad RS [18]	2006	USA	40	PE	Cell block	D2-40	1:50	Signet	Membranous staining
Bhalla R [19]	2007	USA	20	PE,PTE	Cell block	D2-40	1:100	Dako	Membranous staining
Hyun TS [20]	2012	USA	32	PE	Cell block	D2-40	1:50	Dako	Membranous staining
Ordóñez NG [21]	2005	USA	163	Tissue	Tissue section	D2-40	1:50	Signet	Membranous staining
Chu AY [22]	2005	USA	178	Tissue	Tissue block	D2-40	1:25	Signet	Membranous and/or Cytoplasmic staining
Müller AM [23]	2006	Germany	112	Tissue	Tissue section	D2-40	1:50	DCS	Membranous and/or Cytoplasmic staining
Ordóñez NG [24]	2006	USA	85	Tissue	Tissue section	D2-40	1:25	Signet	Membranous staining
Comin CE [25]	2007	Italy	55	Tissue	Surgical resection	D2-40	Pre-diluted	Signet	Membranous staining
Mimura T [26]	2007	Japan	132	Tissue	Major resection	D2-40	1:50	Dako	Membranous staining
Padgett DM [27]	2008	USA	145	Tissue	Surgical resection, autopsy, slides	D2-40	1:100	Signet	Membranous or Cytoplasmic staining (> 10% cells stained, moderate or strong intensity)
Amatya VJ [28]	2009	Japan	160	Tissue	Surgical resection, autopsy	D2-40	Pre-diluted	Nichirei	Membranous and/or Cytoplasmic staining
Deniz H [29]	2009	Turkey	73	Tissue	Tissue section	D2-40	NA	Dako	Membranous staining (> 5% cells stained)
Takeshima Y [30]	2009	Japan	72	Tissue	Surgical section	D2-40	Pre-diluted	Nichirei	Membranous and/or Cytoplasmic staining
Hu YC [31]	2010	USA	67	Tissue	Surgical resection, biopsy	D2-40	1:100	Dako	Membranous and/or Cytoplasmic staining (> 10% cells stained)
Kao SC [32]	2011	Australia	101	Tissue	Surgical section	D2-40	1:100	Signet	Membranous staining
Comin CE [33]	2014	Italy	247	Tissue	Surgical section, biopsy	D2-40	RTU	Ventana	Membranous staining
Guo Z[8]	2016	China	78	Tissue	Biopsy	D2-40	RTU	Dako	NA
Kawai T [34]	2016	Japan	33	Tissue	Biopsy, autopsy, excision	D2-40	RTU	Nichirei	> 10% cells stained
Carbone M [5]	2016	USA	55	Tissue	Biopsy	D2-40	NA	Dako	NA
Amatya VJ [35]	2017	Japan	60	Tissue	Surgical section	D2-40	Pre-diluted	Nichirei	Membranous staining
Kushitani K [36]	2017	Japan	74	Tissue	Biopsy, autopsy, surgical section	D2-40	Pre-diluted	Nichirei	Membranous staining

PE: Pleural effusion; PTE: Peritoneal effusion; NA: Not available; RTU: Ready to use.

**Table 2:** Diagnostic performance, design, and quality of included studies

Author (Ref)	Cases	Controls	TP	FP	FN	TN	Design	Blinded?	QUADAS
Bassarova AV [17]	32	250	32	128	0	122	NA	NA	8
Saad RS [18]	20	20	17	0	3	20	R	Yes	8
Bhalla R [19]	10	10	10	0	0	10	R	NA	9
Hyun TS [20]	11	21	9	0	2	21	R	Yes	10
Ordóñez NG [21]	40	123	29	8	11	115	NA	NA	11
Chu AY [22]	53	125	51	27	2	98	R	NA	12
Müller AM [23]	36	76	22	27	14	49	NA	NA	10
Ordóñez NG [24]	40	45	37	6	3	39	NA	NA	11
Comin CE [25]	15	40	14	8	1	32	R	NA	9
Mimura T [26]	66	66	56	3	10	63	R	NA	11
Padgett DM [27]	44	101	38	4	6	97	R	NA	12
Amatya VJ [28]	80	80	74	13	6	67	R	NA	12
Deniz H [29]	37	36	19	0	18	36	R	NA	11
Takeshima Y [30]	45	27	39	7	6	20	R	NA	10
Hu YC [31]	36	31	25	2	11	29	R	NA	10
Kao SC [32]	80	21	80	0	0	21	R	NA	9
Comin CE [33]	75	172	73	25	2	147	R	NA	10
Guo Z [8]	43	35	38	24	5	11	R	NA	12
Kawai T [34]	22	11	17	3	5	8	P	NA	11
Carbone M [5]	10	45	8	10	2	35	R	NA	10
Amatya VJ [35]	31	29	22	9	9	20	R	Yes	12
Kushitani K [36]	36	38	35	23	1	15	R	NA	11

NA: Not available; P: Prospective; R: Retrospective; FN: False negative; FP: False positive; TN: True negative;

TP: True positive; QUADAS: Quality assessment for diagnostic accuracy studies.

### Diagnostic accuracy of D2-40 immunostaining

**Figure 2:**
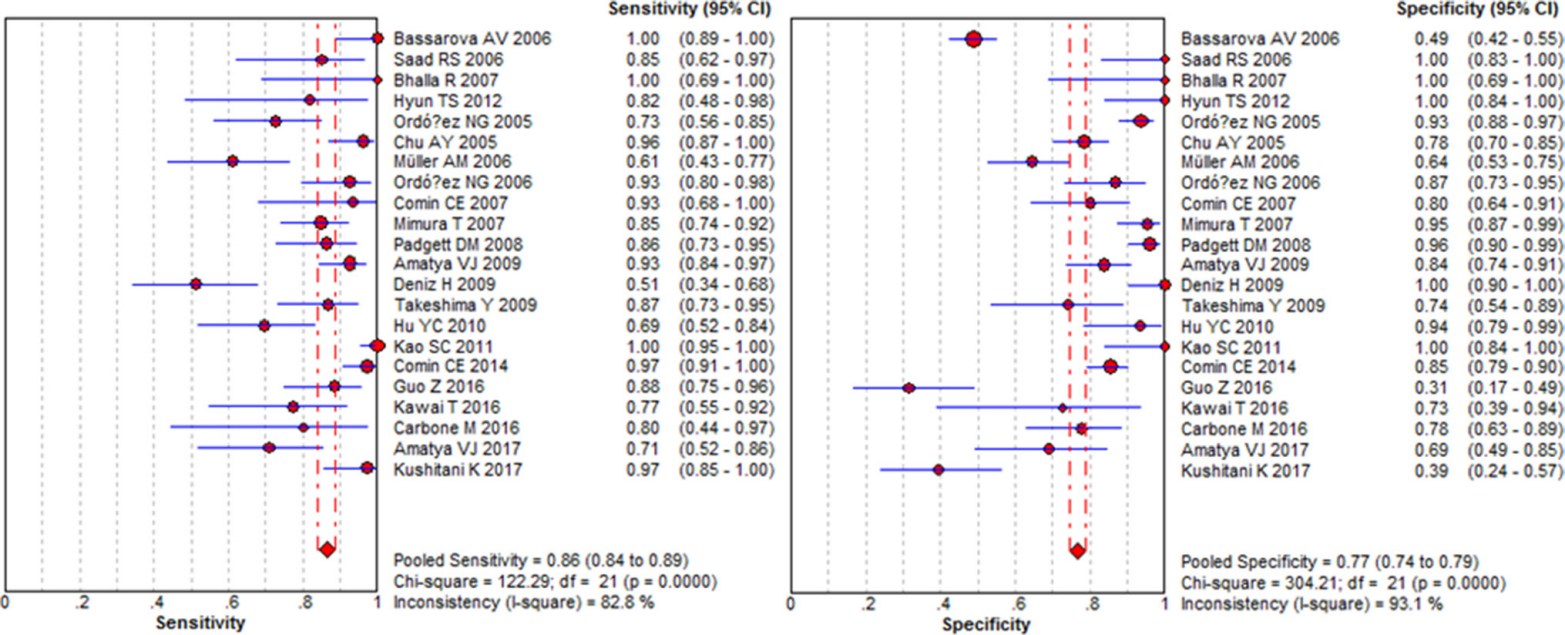
Forest plots of sensitivity and specificity for diagnosing malignant mesothelioma using D2-40 immunostaining

**Figure 3:**
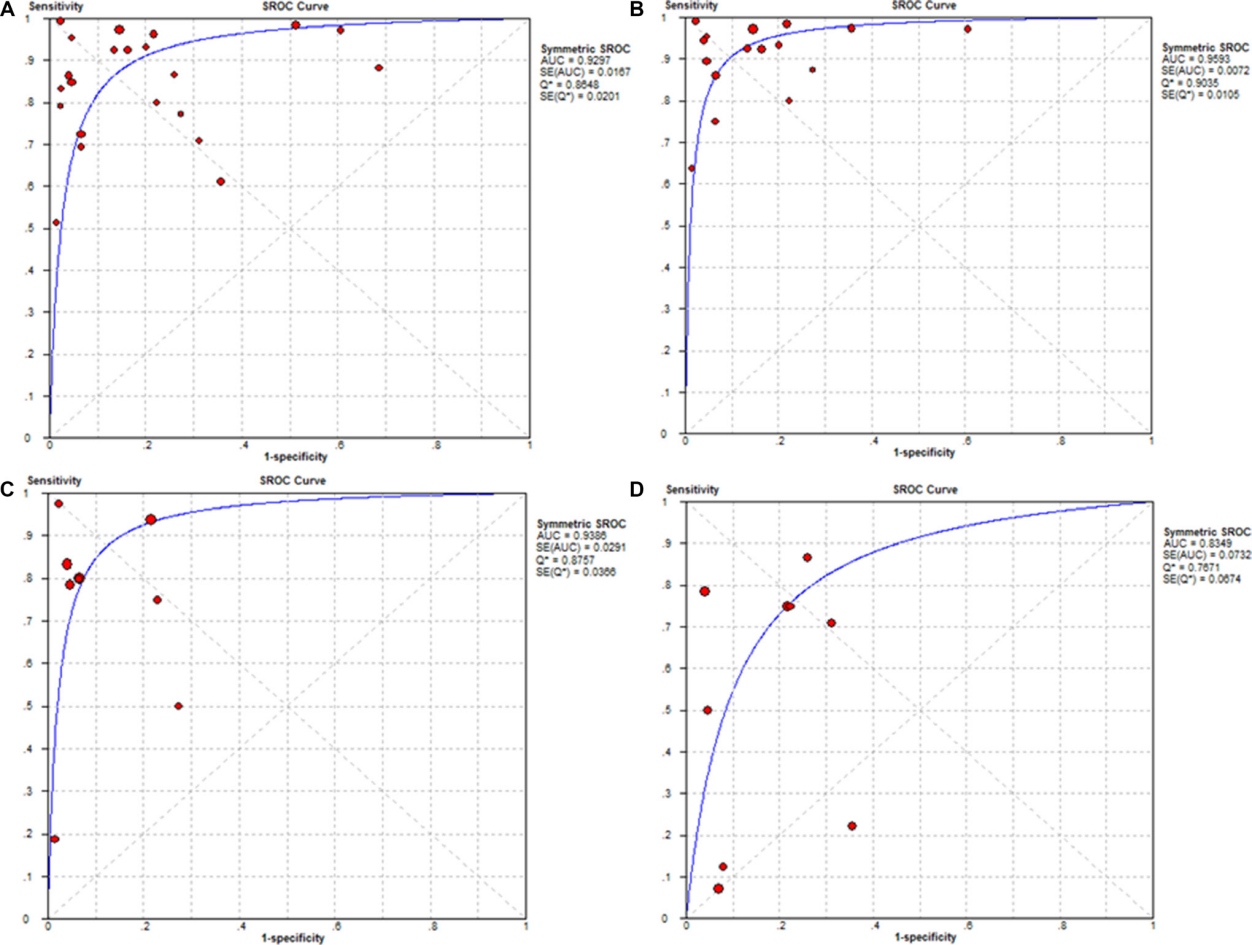
Summary receiver operating characteristic (SROC) curves for diagnosing malignant mesothelioma (MM) and its subtypes using D2-40 immunostaining (**A**) MM (**B**) Epithelioid MM (**C**) Biphasic MM (**D**) Sarcomatoid MM. The SROC curves with confidence and prediction regions around mean operating sensitivity and specificity point analyses of D2-40. AUC, Area under the curve.

The pooled sensitivity and specificity of D2-40 for diagnosing MM was 0.86 (95% CI: 0.84–0.89) and 0.77 (95% CI: 0.74–0.79), respectively (Figure 2). The AUC of SROC curve was 0.93 (Figure 3A). Other parameters of D2-40 for MM were listed in Table 3.

**Table 3:** Accuracy of D2-40 immunostaining for diagnosing malignant mesothelioma (MM)

Carcinoma	No. of studies	Cases	Controls	AUC	SEN [95% CI]	SPE [95% CI]	PLR [95% CI]	NLR [95% CI]	DOR [95% CI]
MM	22	862	1402	0.93	0.86 [0.84, 0.89]	0.77 [0.74, 0.79]	5.15 [3.30, 8.03]	0.18 [0.12, 0.27]	40.37 [19.97, 81.61]
Epithelioid MM	16	540	1020	0.96	0.92 [0.89, 0.94]	0.84 [0.82, 0.87]	6.56 [4.06, 10.58]	0.10 [0.06, 0.18]	85.38 [51.85, 140.60]
Biphasic MM	9	85	559	0.94	0.80 [0.70, 0.88]	0.90 [0.87, 0.92]	8.27 [4.22, 16.21]	0.24 [0.08, 0.70]	43.14 [15.53, 119.82]
Sarcomatoid MM	10	131	659	0.83	0.65 [0.56, 0.73]	0.84 [0.81, 0.87]	3.29 [1.75, 6.16]	0.52 [0.29, 0.92]	6.93 [2.05, 23.47]
Pleural MM	13	536	707	0.93	0.85 [0.82, 0.88]	0.81 [0.78, 0.84]	5.12 [2.94, 8.90]	0.19 [0.11, 0.33]	42.98 [15.21, 121.39]

AUC, Area under the curve; CI: Confidential interval; SEN, Sensitivity; SPE, Specificity; PLR: Positive likelihood ratio; NLR: Negative likelihood ratio; DOR: Diagnostic odds ratio.

After subgroup analysis, diagnostic indices of D2-40 for each subtype of MM (epithelioid, or biphasic, and sarcomatoid) were shown in Figure 3B–3D and Table 3. Parameters of D2-40 for pleural MM were also listed in Table 3.

### Meta-regression

I^2^ values for diagnostic performance indices were as follows: sensitivity, 82.8% (*P* = 0.00); specificity, 93.1% (*P* = 0.00); PLR, 94.2% (*P* = 0.00); NLR, 81.6% (*P* = 0.00); and DOR, 77.3% (*P* = 0.00). These suggested high heterogeneity among included studies. Therefore, a meta-regression analysis was performed based on eight variates: country of origin (USA vs others); sample size (< 100 or ≥ 100); specimen type (effusion vs tissue); D2-40 antibody dilution (< 1:50 vs others); cut-off value (Membranous staining vs others); study design (prospective vs others); and blinding (blind vs Not available); QUADAS score (< 9 vs ≥ 9). None of these covariates was found to be a source of heterogeneity (all *P* ≥ 0.05, Table 4).

**Table 4:** Meta-regression of potential heterogeneity among the included studies

Covariates	No. of studies	Coefficient	SE	RDOR (95% CI)	*P* value
Country					
USA	9	–0.66	0.77	0.52 (0.10–2.76)	0.41
others	13				
Sample size					
< 100	13	0.87	0.70	2.39 (0.52–10.96)	0.24
≥ 100	9				
Specimen					
Effusion	4	–1.32	1.78	0.27 (0.01–13.01)	0.47
Tissue	18				
D2-40 dilution					
< 1:50	5	–1.10	0.96	0.33 (0.04–2.72)	0.27
others	17				
Cut-off					
Membranous staining	13	–1.52	0.72	0.22 (0.05–1.04)	0.05
others	9				
Study Design					
Prospective	1	–0.14	1.60	1.15 (0.04–37.21)	0.93
others	21				
Blinding					
Yes	3	−0.97	1.30	0.38(0.02–6.53)	0.47
NA	19				
QUADAS score					
< 9	2	1.08	2.10	2.95 (0.03–289.83)	0.62
≥ 9	20				

SE: Standard error; RDOR: Relative diagnostic odds ratio; CI: Confidential interval; NA: Not available; QUADAS: Quality assessment for diagnostic accuracy studies.

### Publication bias

**Figure 4:**
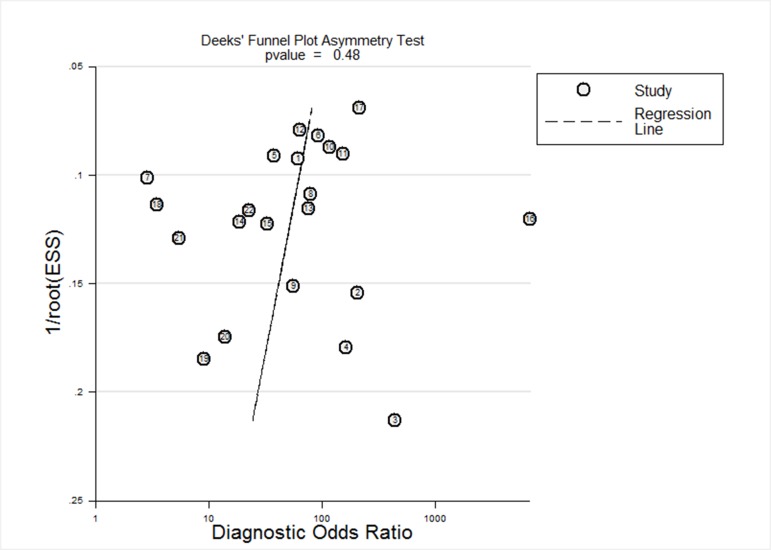
Deeks’ funnel plot to assess the likelihood of publication bias The *P* value of 0.48 for the slope coefficient suggests symmetry in the data and a low likelihood of publication bias.

The slope coefficient of Deeks’ funnel plot was associated with a *P* value of 0.48, suggesting symmetry in the data and low likelihood of such kind of bias among the included studies (Figure 4).

## DISCUSSION

The search for biomarkers, a cost-effective means of MM management, has been on-going for the past years [37]. D2-40 has emerged as a promising candidate, but the results of the studies remain controversial [20, 33, 36]. In our study, we determined the overall accuracy of D2-40 immunostaining in diagnosing MM through a meta-analysis.

Our meta-analysis found the pooled sensitivity and specificity was 0.86 and 0.77 respectively, suggesting a rate of missed diagnoses (14%) and misdiagnosis (23%) of D2-40 for diagnosing MM. The pooled DOR of D2-40 for MM was 40.37, which indicates a modest level of overall accuracy. The pooled PLR value of 5.15 suggests that the patients with MM have an approximately 5-fold higher chance of giving a positive D2-40 result than do patients without MM. At the same time, the pooled NLR was 0.18, indicating that a negative D2-40 result is 18% likely to be a false negative, which is not low enough to rule out MM. Thus, these findings suggested that D2-40 should be combined with other markers to improve the diagnostic accuracy. For example, several studies reported that D2-40 showed low specificity in distinguishing MM from ovarian carcinomas [17, 20, 22]. If the differential diagnosis of peritoneal MM from ovarian carcinomas is required, clinical history of the patient should be an important factor for evaluation. At the same time, the ovarian carcinoma markers, estrogen receptor etc., should be added into the immunohistochemical panel for avoiding a false-positive diagnosis of MM [38].

MM has distinctive histological subtypes: epithelioid (60%∼80%), sarcomatoid (< 10%), and biphasic (10%∼15%, composed of both epithelioid and sarcomatoid components) [39]. Our study further determined the diagnostic performance of D2-40 for these subtypes and found the performance of D2-40 for diagnosing epithelioid MM and biphasic MM is better than that for sarcomatoid MM. It indicated the reactivity to D2-40 antibody differed among these subtypes of MM. A relatively low pooled sensitivity (0.65, 95% CI: 0.56–0.73) for sarcomatoid MM was obtained in our meta-analysis. However, Chirieac LR [40] have reported that all the 24 (100%) cases of sarcomatoid MM were positive for D2-40 staining in their study, which was not included in our meta-analysis for lack of control group. Further work should be performed to elucidate this issue.

In diagnostic practice, pleural MM can easily be confused with metastatic carcinomas with the pleura involvement. Our meta-analysis has performed a subgroup analysis of pleural MM. We found that the sensitivity, specificity, and AUC of D2-40 for diagnosing pleural MM were 0.85, 0.81 and 0.93, respectively. However, interpretation should be with caution when differentiating pleural MM from lung small cell carcinomas (SCC), because it was reported that D2-40 can stained 77% of lung SCC [5]. Recently, novel markers, such as BAP1 [5], MUC4 [35], fibulin-3 [41], have been used to improve the diagnostic accuracy of D2-40 for pleural MM.

Compared with histological diagnosis, cytological diagnosis of MM is minimally invasive and more easily performed. Because only 4 studies using cytology of effusions to diagnose MM can be included in our meta-analysis, the accuracy of diagnosing MM using cytology is hard to be evaluated accurately now. But the difference of biological behavior among the subtypes of MM should be considered when using cytological diagnosis. Epithelioid MM cells readily shed into the pleural or peritoneal space, which can be identified from pleural or peritoneal effusions [42]. While, sarcomatoid MM cells generally do not shed into the pleural or peritoneal space, which leads to poor performance of cytological diagnosis [42]. Thus, cytology-only approach should be not suitable for diagnosing sarcomatoid MM and biphasic MM.

Because both benign and malignant mesothelial cells can react to D2-40 antibody [22, 43], it is needed to distinguish MM from reactive mesothelial hyperplasia in diagnostic practice. Several aspects (such as cellularity, papillae, zonation, growth pattern, vascularity and stromal invasion) should be considered [4, 39, 44]. A new tool, combining molecular data and computational analysis, has been applied for this kind of differential diagnosis [45].

In addition, the operation procedure of D2-40 immunostaining was not uniformed in the included studies. First, though all the included studies used D2-40 clone, its dilution factors ranged from 1:25 to 1:200. Second, cut-off value of positive staining is not consistent among the included studies. According to our experience in D2-40 immunostaining (D2-40 clone, 1:100, Dako, membrane staining), optimal condition, standard operation procedure, and reliable quality control are very important for obtaining satisfactory immunostaining results.

The findings of this meta-analysis should be interpreted with caution because of several limitations. We applied strict inclusion criteria, which may be good for reducing selection bias, but led to only a small set of studies could be included. In addition, we observed the substantial heterogeneity across the included studies, but we were unable to identify its source using meta-regression analysis. Future work should aim to decrease the risk of bias.

## CONCLUSIONS

In summary, our findings indicated that the accuracy of D2-40 immunostaining may be not enough to diagnose MM alone. The results of D2-40 immunostaining should be interpreted in combination with those of other markers. Novel promising markers are also needed to be explored to improve the diagnostic accuracy.

## SUPPLEMENTARY MATERIALS TABLE





## Abbreviations

MM: Malignant Mesothelioma
QUADAS: Quality Assessment for Diagnostic Accuracy Studies
95% CI: 95% Confidence Interval
PLR: Positive Likelihood Ratio
NLR: Negative Likelihood Ratio
DOR: Diagnostic Odds Ratio
SROC: Summary Receiver Operating Characteristic
AUC: Area Under the Curve
SCC: Small Cell Carcinomas
