# Supplementary material for: Diagnostic value of D2-40 immunostaining for malignant mesothelioma: a meta-analysis
Source: Oncotarget. 2017 Jul 6;8(38):64407–16. doi: 10.18632/oncotarget.19041 (PMC5610012; doi:10.18632/oncotarget.19041)
Supplement: Supplementary file 1 [file oncotarget-08-64407-s001.pdf]

Diagnostic value of D2-40 immunostaining for malignant mesothelioma: a meta-analysis

Supplementary Materials

Supplementary Table 1: Characteristics of the controls in included studies. See Supplementary_Table_1
